# The development of a segment-based musculoskeletal model of the lower limb: introducing FreeBody

**DOI:** 10.1098/rsos.140449

**Published:** 2015-06-24

**Authors:** Daniel J. Cleather, Anthony M. J. Bull

**Affiliations:** 1 School of Sport, Health and Applied Sciences, St Mary's University, Twickenham, UK; 2 Department of Bioengineering, Imperial College London, London, UK

**Keywords:** musculoskeletal modelling, muscle force, joint contact force, segment-based approach, joint-based approach, lower limb

## Abstract

Traditional approaches to the biomechanical analysis of movement are joint-based; that is the mechanics of the body are described in terms of the forces and moments acting at the joints, and that muscular forces are considered to create moments about the joints. We have recently shown that segment-based approaches, where the mechanics of the body are described by considering the effect of the muscle, ligament and joint contact forces on the segments themselves, can also prove insightful. We have also previously described a simultaneous, optimization-based, musculoskeletal model of the lower limb. However, this prior model incorporates both joint- and segment-based assumptions. The purpose of this study was therefore to develop an entirely segment-based model of the lower limb and to compare its performance to our previous work. The segment-based model was used to estimate the muscle forces found during vertical jumping, which were in turn compared with the muscular activations that have been found in vertical jumping, by using a Geers' metric to quantify the magnitude and phase errors. The segment-based model was shown to have a similar ability to estimate muscle forces as a model based upon our previous work. In the future, we will evaluate the ability of the segment-based model to be used to provide results with clinical relevance, and compare its performance to joint-based approaches. The segment-based model described in this article is publicly available as a GUI-based Matlab® application and in the original source code (at www.msksoftware.org.uk).

## 1. Introduction

Musculoskeletal modelling technology promises to provide an estimation of the forces experienced by the hard and soft tissues of the body during movement [1–4]. This is important given both the difficulties associated with *in vivo* measurements of these forces and the potential for improved treatment outcomes if the result of an intervention could be simulated *a priori*.

One approach to musculoskeletal modelling of the lower limb is to predict the muscular, ligamentous and joint reaction forces that create movement from measurements of the movement outcome (i.e. the kinematics of the body segments and the ground reaction force (GRF)), an approach which is referred to as inverse dynamics. Traditional approaches to inverse dynamics tend to pose the equations of motion in terms of the forces and moments that act across the joints. This simplifies the description of motion by making it unnecessary to explicitly describe all the forces and moments experienced by the limb which, instead, are implicitly captured by the concept of the joint. The alternative to this ‘joint-based’ approach is to pose the equations of motion in terms of the forces and moments that act upon the body segments (a ‘segment-based’ approach) which requires all the forces and moments to be explicitly described. We have recently demonstrated that segment-based approaches can provide additional insights as to the functional anatomy of the lower limb [5,6], not least because of the additional detail that is incorporated within the approach. However, these analyses have been based on the consideration of simple two-dimensional models and not a detailed three-dimensional (3D) model of the lower limb.

To date, few 3D segment-based models have been proposed within the literature (notable exceptions being the lower limb model of Moissenet *et al.* [7] and the upper limb model of Pennestri *et al.* [8]). In our previous work, we have described a detailed, 3D, optimization-based approach to inverse dynamics analysis which we have argued has advantages over traditional approaches [9–11] (these findings have been supported by other groups [7,12]). However, this model is not entirely segment-based in its approach. In particular, a number of joint-based assumptions are made in order to simplify the modelling of the knee. The purpose of this study was therefore to develop a fully segment-based model of the lower limb and to compare its predictions to our previous work. Finally, the model described within this paper has been made publicly available, and this is also described towards the end of this paper.

## 2. Material and methods

This study describes the development of a new musculoskeletal model of the lower limb that is based upon our previous work [9–11,13]. The lower limb is modelled as a 3D arrangement of five rigid segments that represent the foot, shank, thigh, pelvis and patella (the patella segment is assumed to have zero mass). The data inputted into the model consists of motion capture data describing the position of retro-reflective markers attached to a subject (kinematic data) and ground reaction forces (kinetic data). The motion capture data are used to specify the position and orientation of the segments as a function of time, and from this the kinematics of the segments can be calculated. The model is then used to estimate the muscular and joint reaction forces experienced by the lower limb during the recorded movement. The forces that create the observed movement are therefore predicted from their resultant movements and forces (inverse dynamics analysis).

### 2.1 Model posture

**Figure 1.**
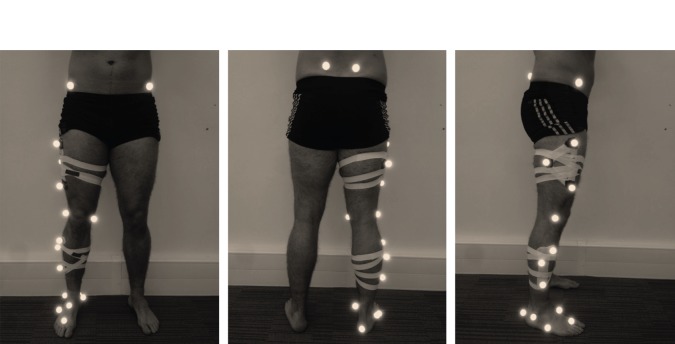
Position of markers on a subject standing in the calibration (anatomical) position.

The locations and orientations of the foot, shank, thigh and pelvis segments are derived from raw data that describe the location (in the laboratory fixed global coordinate frame; GCS) of retro-reflective markers placed upon key anatomical landmarks (table 1). Each segment has a segment fixed local coordinate frame (LCS) which is defined by reference to the position of the markers when the subject is stood in a known calibration position (the anatomical position; figure 1). Within each LCS, the *y*-axis is first defined to run from the distal joint to the proximal joint. An intermediate *z*-axis from medial to lateral is then defined by reference to anatomical landmarks, and then the *x*-axis found such that it is orthogonal to the *y*- and intermediate *z*-axes. Finally, the permanent *z*-axis is found such that it is orthogonal to the *x*- and *y*-axes. The origin of each LCS is located at the centre of the proximal joint (apart from the pelvis, whose origin is at the midpoint of a line connecting the anterior superior iliac spines). In constructing the LCS of each segment, the transformation of the segment from the LCS to the calibration position is found. Next, the transformation of each segment from the calibration position to its position in each frame is found using the method of Horn [14]. Ultimately, the location and orientation of a segment for each frame is defined by the transformation from the LCS of the segment to its position in the GCS (this can be found by combining the former two transformations). Within the GCS, the *x*-axis runs from posterior to anterior, the *y*-axis from inferior to superior and the *z*-axis from medial to lateral.

**Table 1.** Marker positions used for data capture.

marker	location
FCC	calcaneus
FMT	tuberosity of the fifth metatarsal
FM2	head of the second metatarsal
TF	additional marker placed on the foot
FAM	apex of the lateral malleolus
TAM	apex of the medial malleolus
C1, C2, C3	additional markers placed on the shank segment
FLE	lateral femoral epicondyle
FME	medial femoral epicondyle
T1, T2, T3	additional markers placed on the thigh segment
RASIS	right anterior superior iliac spine
LASIS	left anterior superior iliac spine
RPSIS	right posterior superior iliac spine
LPSIS	left posterior superior iliac spine

### 2.2 Patellar posture

The position and orientation of the patella is given as a function of the tibiofemoral joint flexion angle (*θ*) using a new model of the patella that has been developed from the literature as follows. First, the location of the patella origin is established relative to the position of the tibial tuberosity in the tibial LCS (this is taken from the data of Klein Horsman *et al.* [15] and scaled in the same way as is described later for the other muscle parameters). This is achieved by determining the path of the patellar tendon and by defining the origin of the patella to be at the point where the patellar tendon meets the patella. Specifically, the orientation of the patellar tendon relative to the tibial LCS can be described by the combination of the patellar tendon sagittal and coronal plane angles. These are calculated from the knee flexion angle using polynomial regression equations derived from the results of Kobayashi *et al.* [16] (table 2). Combining these angles with the length of the patellar tendon (which is taken from Klein Horsman *et al.* and scaled to the subject characteristics) allows the position of the patella origin to be found.

**Table 2.** Coefficients describing the position and orientation of the patella as a function of knee flexion angle. (The applicable equation is of the form: variable= *b*_0_+*b*_1_*θ*+*b*_2_*θ*^2^+*b*_3_*θ*^3^+*b*_4_*θ*^4^, where *θ* is the knee flexion angle. PT, patellar tendon.)

variable	*b*_0_	*b*_1_	*b*_2_	*b*_3_	*b*_4_
PT sagittal plane angle	20.4	−2.60×10^−1^			
PT coronal plane angle	10.9	−2.33×10^−1^	1.89×10^−3^	−5.69×10^−6^	
patellar flexion	5.59	6.60×10^−1^			
patellar tilt	1.63	6.67×10^−2^	1.44×10^−4^	−5.37×10^−6^	
patellar rotation	1.43	1.06×10^−1^	−3.45×10^−3^	5.47×10^−5^	−2.38×10^−7^

Next, the orientation of the patella is established, again by reference to the knee flexion angle. The orientation of the patella is given based upon a set of three Euler angles. The sequence of rotation is as follows: (i) patellar flexion about the *z*-axis (of the femoral LCS); (ii) patellar tilt about the new *y*′ axis; and (iii) patellar rotation about the new *x*′′ axis. These three angles are also calculated using polynomial regression equations, this time developed from the data of Nha *et al.* [17]

The above calculations allow the transformation from the patellar LCS to the GCS to be established. This in turn means that the position and orientation of each segment for each time point has been established. Note that anatomical landmarks were used to combine the above datasets in order to ensure their compatibility.

### 2.3 Adding the musculoligamentous geometry

Once the locations and orientations of every segment for every frame have been determined, it is then possible to add the next layer of detail—that is the geometry of the muscular and ligamentous structures. This is achieved by using the data of Klein Horsman *et al.* [15] which specify the origin, insertion and path of 163 muscle elements and 14 ligaments. The data of Klein Horsman *et al.* is used to define the locations of the origins and insertions of the muscles and ligaments within the LCS of the applicable segments (this is achieved by constructing an LCS for each of the Klein Horsman *et al.* segments using exactly the same methodology as is used to define the LCS of the subject). The geometry of the musculoligamentous system can then be calculated for each frame using the transformations from LCS to GCS described earlier.

### 2.4 Scaling

The musculoskeletal geometry of Klein Horsman *et al.* [15] is scaled to match the size of the subject under consideration. This is achieved at the segmental level, by calculating the relative size of the subject's segments in comparison to that of the Klein Horsman cadaver, and then adjusting the coordinates of origins and insertions accordingly (linearly within the Cartesian 3D frame).

### 2.5 Wrapping

For muscle and ligament elements that do not wrap around other musculoskeletal structures, their line of action is taken to be the straight line from origin to insertion. For those elements that do wrap around other structures, the wrapping of the element is represented in one of two ways. For the majority of the muscles, the wrapping is described by the description of additional ‘via’ points through which the path of the muscle is constrained to pass, and that thus define the line of action of the muscle. These via points are also taken from the work of Klein Horsman *et al.* However, in the case where the muscle element is free to move over the underlying surface, a wrapping cylinder is defined to represent the underlying structures, and then the path of the element around the cylinder is found using the method of Charlton & Johnson [18]. The wrapping cylinders are also taken from the work of Klein Horsman *et al.*, however, an additional cylinder was also defined to represent the wrapping of the quadriceps tendon around the femoral condyles at deeper knee flexion angles [19].

### 2.6 Anthropometry and kinematics

The anthropometry of the model (i.e. the mass, centre of mass and inertia tensor of each segment) is generated using the data of de Leva [20]. The kinematics of the movement (i.e. the linear and angular velocities and accelerations of each segment) is calculated using the quaternion-based methodology of Dumas *et al.* [21].

### 2.7 Calculating the model kinetics

The analysis approach used in this study follows the example of our previous work [9–11]. That is, the indeterminate equations of motion governing the movement of the lower limb (in the GCS) are posed based upon the musculoskeletal geometry and the measured kinematics (segment motions) and kinetics (ground reaction forces) of the lower limb. An optimization approach is then used to solve the equations of motion simultaneously. In this study, a number of different formulations of the equations of motion are employed (table 3), in order to evaluate the effect of transitioning to a segment-based model and adding more detail relating to the geometry of the knee joint, and these are described below. It should be noted that in all cases the method of Dumas *et al.* [21] is used to formulate the equations of motion.

**Table 3.** Details of the different cases considered in this study.

case	patellofemoral joint modelled?	two tibiofemoral joint contacts?	joint reaction forces explicit?
1	no	no	no
2	yes	no	no
3	yes	no	yes
4	yes	yes	yes

Case 1 comprises the same solution approach as presented in our previous work [11], although using the revised musculoskeletal geometry described above. First, the inter-segmental forces can be found directly by the iterative application of Newton's second law to each segment, working from distal to proximal:

2.1 $${-\hat{S}}^{k}=m^{k}({\hat{a}}^{k}-\hat{g})-{\hat{S}}^{k-1}.$$

Next, the equations of motion are posed by considering the motion of foot, shank and thigh segments. This allows 18 indeterminate equations of motion to be written (equation (2.2); table 4)—nine describing the linear motion of the segments (three for each segment) and nine describing the rotational motion (again three for each segment). The equations of motion are parametrized by the previously calculated musculoskeletal geometry, the kinematics of the segments and the GRF. The 186 unknowns consist of the muscular, ligamentous and joint reaction forces (bone on bone contact forces) experienced by the lower limb. It should be noted that each segment has a full complement of 6 degrees of freedom (d.f.) and there are no constraints imposed based upon considerations of the kinematics of the joints (with the exception of the patellofemoral joint). This means that each joint effectively also has 6 d.f. (apart from the patellofemoral joint), although the joints are not explicitly described in the model:

2.2 $$\begin{array}{rl} & \left(\begin{array}{cccccc} {\hat{p}}_{1}^{1}\cdots {\hat{p}}_{M}^{1} & \;{\hat{p}}_{\mathrm{pt}}^{1} & \;{\hat{q}}_{1}^{1}\cdots {\hat{q}}_{N}^{1} & {-I}_{3\times 3} & E_{3\times 3} & E_{3\times 3}\\ {\hat{p}}_{1}^{2}\cdots {\hat{p}}_{M}^{2} & \;{\hat{p}}_{\mathrm{pt}}^{2} & \;{\hat{q}}_{1}^{2}\cdots {\hat{q}}_{N}^{2} & I_{3\times 3} & {-I}_{3\times 3} & E_{3\times 3}\\ {\hat{p}}_{1}^{3}\cdots {\hat{p}}_{M}^{3} & \;{\hat{p}}_{\mathrm{pt}}^{3} & \;{\hat{q}}_{1}^{3}\cdots {\hat{q}}_{N}^{3} & E_{3\times 3} & I_{3\times 3} & {-I}_{3\times 3}\\ {\hat{v}}_{1}^{1}\cdots {\hat{v}}_{M}^{1} & \;{\hat{v}}_{\mathrm{pat}}^{1} & \;{\hat{w}}_{1}^{1}\cdots {\hat{w}}_{N}^{1} & E_{3\times 3} & E_{3\times 3} & E_{3\times 3}\\ {\hat{v}}_{1}^{2}\cdots {\hat{v}}_{M}^{2} & \;{\hat{v}}_{\mathrm{pat}}^{2} & \;{\hat{w}}_{1}^{2}\cdots {\hat{w}}_{N}^{2} & E_{3\times 3} & E_{3\times 3} & E_{3\times 3}\\ {\hat{v}}_{1}^{3}\cdots {\hat{v}}_{M}^{3} & \;{\hat{v}}_{\mathrm{pat}}^{3} & \;{\hat{w}}_{1}^{3}\cdots {\hat{w}}_{N}^{3} & E_{3\times 3} & E_{3\times 3} & E_{3\times 3} \end{array}\right)\left(\begin{array}{c} F_{1}\\ \vdots \\ F_{M}\\ F_{\mathrm{pt}}\\ L_{1}\\ \vdots \\ L_{N}\\ {Rx}^{1}\\ {Ry}^{1}\\ {Rz}^{1}\\ \vdots \\ {Rx}^{3}\\ {Ry}^{3}\\ {Rz}^{3} \end{array}\right)\\ & \;=\left(\begin{array}{c} m^{1}({\hat{a}}^{1}-\hat{g})-{\hat{S}}^{0}\\ m^{2}({\hat{a}}^{2}-\hat{g})\\ m^{3}({\hat{a}}^{3}-\hat{g})\\ m^{1}{\hat{c}}^{1}\times ({\hat{a}}^{1}-\hat{g})+Y_{3\times 3}^{1}{\ddot{\hat{\varphi }}}^{1}+{\dot{\hat{\varphi }}}^{1}\times Y_{3\times 3}^{1}{\dot{\hat{\varphi }}}^{1}-{\hat{d}}^{1}\times {\hat{S}}^{0}-{\hat{W}}^{0}\\ m^{2}{\hat{c}}^{2}\times ({\hat{a}}^{2}-\hat{g})+Y_{3\times 3}^{2}{\ddot{\hat{\varphi }}}^{2}+{\dot{\hat{\varphi }}}^{2}\times Y_{3\times 3}^{2}{\dot{\hat{\varphi }}}^{2}-{\hat{d}}^{2}\times {\hat{S}}^{1}\\ m^{3}{\hat{c}}^{3}\times ({\hat{a}}^{3}-\hat{g})+Y_{3\times 3}^{3}{\ddot{\hat{\varphi }}}^{3}+{\dot{\hat{\varphi }}}^{3}\times Y_{3\times 3}^{3}{\dot{\hat{\varphi }}}^{3}-{\hat{d}}^{3}\times {\hat{S}}^{2} \end{array}\right). \end{array}$$

In equation (2.2), the rotational effect of each muscle and ligament element upon a segment *k* is captured by calculating an effective moment arm (${\hat{v}}_{i}^{k}$ and ${\hat{w}}_{j}^{k}$, respectively) which describe the overall rotational effect of 1 N of tension in the element on the given segment [5]. So for a monoarticular muscle, ${\hat{v}}_{i}^{k}$ includes the effect of both the muscle force and the joint reaction force that arises because of the muscle force. This is akin to the assumptions made in a joint-based approach. Equally, for the intermediate segment of a biarticular muscle that does not attach to the segment, ${\hat{v}}_{i}^{k}$ captures the rotational effects of the joint reaction forces created by tension in the muscle [10].

**Table 4.** Nomenclature used in equations of motion.

${\hat{a}}^{k}$	linear acceleration of the centre of mass of segment *k*
${\hat{c}}^{k}$	vector from centre of rotation of joint at proximal end of segment *k* to centre of mass of segment *k*
${\hat{d}}^{k}$	vector from centre of rotation of joint at proximal end of segment *k* to centre of rotation joint at distal end of segment *k*
${\tilde{d}}^{k}$	skew-symmetric matrix of vector
${\tilde{d}}_{l}^{3}$	skew-symmetric matrix of vector from centre of rotation of hip to tibiofemoral joint contact *l*
*E*_3×3_	3×3 matrix of zeros
${\tilde{f}}^{3}$	skew-symmetric matrix of vector from centre of rotation of hip to contact point of patella with the femur
*F*_*i*_	magnitude of force in muscle *i*
$F_{\max_{i}}$	maximum possible force in muscle *i* (upper bound)
$\hat{g}$	acceleration due to gravity
${\tilde{h}}_{l}^{2}$	skew-symmetric matrix of vector from centre of rotation of knee to tibiofemoral joint contact *l*
*i*	muscle number
*I*_3×3_	3×3 identity matrix
*j*	ligament number
*J*	cost function
*k*	segment number
*L*_*j*_	magnitude of force in ligament *j*
$L_{\max_{j}}$	maximum possible force in ligament *j* (upper bound)
*m*^*k*^	mass of segment *k*
*M*	total number of muscles
*N*	total number of ligaments
${\hat{p}}_{i}^{k}$	unit vector representing the line of action of force created by muscle *i* that acts on segment *k* (zero if muscle does not insert on segment *k*)
pat	patella
pt	patellar tendon
*P*/*Q* ratio	ratio of patellar tendon to quadriceps tendon force
${\hat{q}}_{j}^{k}$	unit vector representing the line of action of force created by ligament *j* that acts on segment *k* (zero if ligament does not insert on segment *k*)
${\hat{r}}_{i}^{k}$	vector from centre of rotation of joint at proximal end of segment *k* to point of action of muscle *i* on segment *k* (zero if muscle does not insert on segment *k*)
*Rx*^*k*^	*x* component of reaction force acting at proximal end of segment *k*
*Ry*^*k*^	*y* component of reaction force acting at proximal end of segment *k*
*Rz*^*k*^	*z* component of reaction force acting at proximal end of segment *k*
${\hat{R}}^{k}$	vector representing *x*, *y* and *z* components of reaction force acting at proximal end of segment *k*
${\hat{R}}_{l}^{k}$	vector representing *x*, *y* and *z* components of reaction force *l* acting at proximal end of segment *k*
${\hat{s}}_{j}^{k}$	vector from centre of rotation of joint at proximal end of segment *k* to point of action of ligament *j* on segment *k* (zero if ligament does not insert on segment *k*)
${-\hat{S}}^{k}$	inter-segmental force acting on proximal end of segment *k*
${\hat{v}}_{i}^{k}$	effective moment arm of muscle *i* on segment *k*
${\hat{w}}_{j}^{k}$	effective moment arm of ligament *j* on segment *k*
${-\hat{W}}^{k}$	inter-segmental moment acting on proximal end of segment *k*
$Y_{3\times 3}^{k}$	inertia tensor of segment *k*
*ρ*_*i*_	*P*/*Q* ratio for muscle *i* (zero if the muscle is not part of the quadriceps muscle group)
${\dot{\hat{\varphi }}}^{k}$	angular velocity of segment *k*
${\ddot{\hat{\varphi }}}^{k}$	angular acceleration of segment *k*

**Figure 2.**
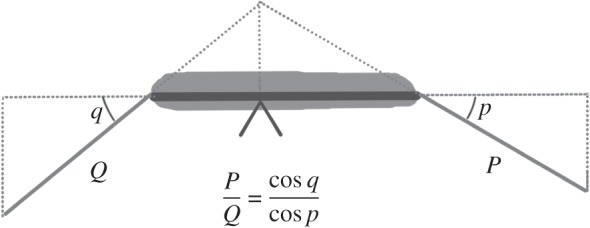
Moment equilibrium of the patella results in a changing ratio between the patellar and quadriceps tendon forces (*P* and *Q*, respectively) which depends on the angles of incidence of the two tendons on the patella (*p* and *q*, respectively).

In case 1, the effect of tension in the patellar tendon is assumed to create an equal and opposite effect on the tibial and femoral segments, and the patellofemoral joint is not explicitly modelled (this is again a common assumption in a joint-based approach). However, the patella is assumed to be in force and moment equilibrium and thus the *P*/*Q* ratio (the ratio of the patellar to quadriceps tendon forces) will alter with knee flexion [22]. The *P*/*Q* ratio is derived from a consideration of the musculoskeletal geometry of the patella in the sagittal plane (figure 2) and then the effective force upper bound of the patellar tendon is adjusted as a function of the knee flexion angle to reflect this relationship.

In case 2, an explicit consideration of the patella is used to derive the equations of motion. First, there is the addition of three more unknowns representing the *x*, *y* and *z* components of the patellofemoral joint reaction force. The patellofemoral joint reaction force is included in the equations of motion of the thigh segment, and in addition, the lines of action of the quadriceps muscle elements are explicitly modelled. Second, three more equations of motion are added that describe the force equilibrium of the patella. Finally, a fourth equation of motion is added which ensures that the sagittal plane components of the patellar tendon and quadriceps forces are in the same ratio as that which was derived from geometric considerations in case 1. It should be noted that the patella is also assumed to be in moment equilibrium but this assumption is not explicitly captured in the equations of motion. Instead, the pivot point of the patella is assumed to shift in order to maintain equilibrium. The patellofemoral joint contact position is found for each frame by finding this pivot point, which is in turn used to define the contact position between the patella and thigh segments.

The equations of motion in case 2 (equation (2.3); table 4) thus consist of 21 equations and 190 unknowns (three additional unknowns representing the patellofemoral joint reaction force and one additional unknown representing the patellar tendon force which is now modelled separately from the quadriceps components):

2.3 $$\begin{array}{rl} & \left(\begin{array}{ccccccc} {\hat{p}}_{1}^{1}\cdots {\hat{p}}_{M}^{1} & \;{\hat{p}}_{\mathrm{pt}}^{1} & \;{\hat{q}}_{1}^{1}\cdots {\hat{q}}_{N}^{1} & {-I}_{3\times 3} & E_{3\times 3} & E_{3\times 3} & E_{3\times 3}\\ {\hat{p}}_{1}^{2}\cdots {\hat{p}}_{M}^{2} & \;{\hat{p}}_{\mathrm{pt}}^{2} & \;{\hat{q}}_{1}^{2}\cdots {\hat{q}}_{N}^{2} & I_{3\times 3} & {-I}_{3\times 3} & E_{3\times 3} & E_{3\times 3}\\ {\hat{p}}_{1}^{3}\cdots {\hat{p}}_{M}^{3} & \;{\hat{p}}_{\mathrm{pt}}^{3} & \;{\hat{q}}_{1}^{3}\cdots {\hat{q}}_{N}^{3} & E_{3\times 3} & I_{3\times 3} & {-I}_{3\times 3} & I_{3\times 3}\\ {\hat{v}}_{1}^{1}\cdots {\hat{v}}_{M}^{1} & \;{\hat{v}}_{\mathrm{pat}}^{1} & \;{\hat{w}}_{1}^{1}\cdots {\hat{w}}_{N}^{1} & E_{3\times 3} & E_{3\times 3} & E_{3\times 3} & E_{3\times 3}\\ {\hat{v}}_{1}^{2}\cdots {\hat{v}}_{M}^{2} & \;{\hat{v}}_{\mathrm{pat}}^{2} & \;{\hat{w}}_{1}^{2}\cdots {\hat{w}}_{N}^{2} & E_{3\times 3} & E_{3\times 3} & E_{3\times 3} & E_{3\times 3}\\ {\hat{v}}_{1}^{3}\cdots {\hat{v}}_{M}^{3} & \;{\hat{v}}_{\mathrm{pat}}^{3} & \;{\hat{w}}_{1}^{3}\cdots {\hat{w}}_{N}^{3} & E_{3\times 3} & E_{3\times 3} & E_{3\times 3} & {\tilde{f}}^{3}\\ {\hat{p}}_{1}^{\mathrm{pat}}\cdots {\hat{p}}_{M}^{\mathrm{pat}} & \;{\hat{p}}_{\mathrm{pt}}^{\mathrm{pat}} & \;E_{3\times N} & E_{3\times 3} & E_{3\times 3} & E_{3\times 3} & {-I}_{3\times 3}\\ \rho_{1}\cdots \rho_{M} & \;-1 & \;E_{1\times N} & E_{1\times 3} & E_{1\times 3} & E_{1\times 3} & E_{1\times 3} \end{array}\right)\;\left(\begin{array}{c} F_{1}\\ \vdots \\ F_{M}\\ F_{\mathrm{pt}}\\ L_{1}\\ \vdots \\ L_{N}\\ {Rx}^{1}\\ {Ry}^{1}\\ {Rz}^{1}\\ \vdots \\ {Rx}^{3}\\ {Ry}^{3}\\ {Rz}^{3}\\ {Rx}^{\mathrm{pat}}\\ {Ry}^{\mathrm{pat}}\\ {Rz}^{\mathrm{pat}} \end{array}\right)\\ & \;=\left(\begin{array}{c} m^{1}({\hat{a}}^{1}-\hat{g})-{\hat{S}}^{0}\\ m^{2}({\hat{a}}^{2}-\hat{g})\\ m^{3}({\hat{a}}^{3}-\hat{g})\\ m^{1}{\hat{c}}^{1}\times ({\hat{a}}^{1}-\hat{g})+Y_{3\times 3}^{1}{\ddot{\hat{\varphi }}}^{1}+{\dot{\hat{\varphi }}}^{1}\times Y_{3\times 3}^{1}{\dot{\hat{\varphi }}}^{1}-{\hat{d}}^{1}\times {\hat{S}}^{0}-M^{0}\\ m^{2}{\hat{c}}^{2}\times ({\hat{a}}^{2}-\hat{g})+Y_{3\times 3}^{2}{\ddot{\hat{\varphi }}}^{2}+{\dot{\hat{\varphi }}}^{2}\times Y_{3\times 3}^{2}{\dot{\hat{\varphi }}}^{2}-{\hat{d}}^{2}\times {\hat{S}}^{1}\\ m^{3}{\hat{c}}^{3}\times ({\hat{a}}^{3}-\hat{g})+Y_{3\times 3}^{3}{\ddot{\hat{\varphi }}}^{3}+{\dot{\hat{\varphi }}}^{3}\times Y_{3\times 3}^{3}{\dot{\hat{\varphi }}}^{3}-{\hat{d}}^{3}\times {\hat{S}}^{2}\\ E_{3\times 1}\\ 0 \end{array}\right). \end{array}$$

For cases 1 and 2, the joint reaction forces (apart from the patellofemoral joint reaction force in case 2) are not described explicitly in the moment parts of the equations of motion. The approach is therefore a hybrid between joint- and segment-based approaches. In case 3, the joint reaction forces are also explicitly included in the moment parts of the equation of motion (equation (2.4); table 4). Otherwise, case 3 is the same as case 2. The position at which the joint reaction forces are considered to act is defined in the following way. The joint contact points are assumed to be the joint centres given in the Klein Horsman *et al.* [15] dataset. These are defined to be fixed within the distal segment of a joint—thus the contact position on the proximal segment is not fixed, and will change if there is a translation of the two segments relative to one another. The contact surfaces are assumed to be rigid in this model:

2.4 $$\begin{array}{rl} & \left(\begin{array}{ccccccc} {\hat{p}}_{1}^{1}\cdots {\hat{p}}_{M}^{1} & \;{\hat{p}}_{\mathrm{pt}}^{1} & \;{\hat{q}}_{1}^{1}\cdots {\hat{q}}_{N}^{1} & {-I}_{3\times 3} & E_{3\times 3} & E_{3\times 3} & E_{3\times 3}\\ {\hat{p}}_{1}^{2}\cdots {\hat{p}}_{M}^{2} & \;{\hat{p}}_{\mathrm{pt}}^{2} & \;{\hat{q}}_{1}^{2}\cdots {\hat{q}}_{N}^{2} & I_{3\times 3} & {-I}_{3\times 3} & E_{3\times 3} & E_{3\times 3}\\ {\hat{p}}_{1}^{3}\cdots {\hat{p}}_{M}^{3} & \;{\hat{p}}_{\mathrm{pt}}^{3} & \;{\hat{q}}_{1}^{3}\cdots {\hat{q}}_{N}^{3} & E_{3\times 3} & I_{3\times 3} & {-I}_{3\times 3} & I_{3\times 3}\\ {{\hat{r}}_{1}^{1}\times \hat{p}}_{1}^{1}\cdots {{\hat{r}}_{M}^{1}\times \hat{p}}_{M}^{1} & \;{{\hat{r}}_{\mathrm{pt}}^{1}\times \hat{p}}_{\mathrm{pt}}^{1} & \;{{\hat{s}}_{1}^{1}\times \hat{q}}_{1}^{1}\cdots {{\hat{s}}_{N}^{1}\times \hat{q}}_{N}^{1} & E_{3\times 3} & E_{3\times 3} & E_{3\times 3} & E_{3\times 3}\\ {{\hat{r}}_{1}^{2}\times \hat{p}}_{1}^{2}\cdots {{\hat{r}}_{M}^{2}\times \hat{p}}_{M}^{2} & \;{{\hat{r}}_{\mathrm{pt}}^{2}\times \hat{p}}_{\mathrm{pt}}^{2} & \;{{\hat{s}}_{1}^{2}\times \hat{q}}_{1}^{2}\cdots {{\hat{s}}_{N}^{2}\times \hat{q}}_{N}^{2} & {\tilde{d}}^{2} & E_{3\times 3} & E_{3\times 3} & E_{3\times 3}\\ {{\hat{r}}_{1}^{3}\times \hat{p}}_{1}^{3}\cdots {{\hat{r}}_{M}^{3}\times \hat{p}}_{M}^{3} & \;{{\hat{r}}_{\mathrm{pt}}^{3}\times \hat{p}}_{\mathrm{pt}}^{3} & \;{{\hat{s}}_{1}^{3}\times \hat{q}}_{1}^{3}\cdots {s_{N}^{3}\times \hat{q}}_{N}^{3} & E_{3\times 3} & {\tilde{d}}^{3} & E_{3\times 3} & {\tilde{f}}^{3}\\ {\hat{p}}_{1}^{\mathrm{pat}}\cdots {\hat{p}}_{M}^{\mathrm{pat}} & \;{\hat{p}}_{\mathrm{pt}}^{\mathrm{pat}} & \;E_{3\times N} & E_{3\times 3} & E_{3\times 3} & E_{3\times 3} & {-I}_{3\times 3}\\ \rho_{1}\cdots \rho_{M} & \;-1 & \;E_{1\times N} & E_{1\times 3} & E_{1\times 3} & E_{1\times 3} & E_{1\times 3} \end{array}\right)\left(\begin{array}{c} F_{1}\\ \vdots \\ F_{M}\\ F_{\mathrm{pt}}\\ L_{1}\\ \vdots \\ L_{N}\\ {Rx}^{1}\\ {Ry}^{1}\\ {Rz}^{1}\\ \vdots \\ {Rx}^{3}\\ {Ry}^{3}\\ {Rz}^{3}\\ {Rx}^{\mathrm{pat}}\\ {Ry}^{\mathrm{pat}}\\ {Rz}^{\mathrm{pat}} \end{array}\right)\\ & \;=\left(\begin{array}{c} m^{1}({\hat{a}}^{1}-\hat{g})-{\hat{S}}^{0}\\ m^{2}({\hat{a}}^{2}-\hat{g})\\ m^{3}({\hat{a}}^{3}-\hat{g})\\ m^{1}{\hat{c}}^{1}\times ({\hat{a}}^{1}-\hat{g})+Y_{3\times 3}^{1}{\ddot{\hat{\varphi }}}^{1}+{\dot{\hat{\varphi }}}^{1}\times Y_{3\times 3}^{1}{\dot{\hat{\varphi }}}^{1}-{\hat{d}}^{1}\times {\hat{S}}^{0}-M^{0}\\ m^{2}{\hat{c}}^{2}\times ({\hat{a}}^{2}-\hat{g})+Y_{3\times 3}^{2}{\ddot{\hat{\varphi }}}^{2}+{\dot{\hat{\varphi }}}^{2}\times Y_{3\times 3}^{2}{\dot{\hat{\varphi }}}^{2}\\ m^{3}{\hat{c}}^{3}\times ({\hat{a}}^{3}-\hat{g})+Y_{3\times 3}^{3}{\ddot{\hat{\varphi }}}^{3}+{\dot{\hat{\varphi }}}^{3}\times Y_{3\times 3}^{3}{\dot{\hat{\varphi }}}^{3}\\ E_{3\times 1}\\ 0 \end{array}\right). \end{array}$$

In case 4, the tibiofemoral joint contact force is compartmentalized into a medial and a lateral component (equation (2.5); table 4). The number of unknowns representing the tibiofemoral joint contact force is thus increased from 3 (*x*, *y* and *z* components) to 6. Thus, the number of unknowns for case 4 is 193.

For cases 1–3 the joint reaction forces are considered to act through the centres of rotation of the joints taken from the Klein Horsman *et al.* dataset [15]. In case 4, the lateral and medial tibiofemoral contact forces act through points 2 cm laterally and medially to the centre of rotation (an inter-condyle distance of 4 cm was chosen following the example of previous work [23]). The tibiofemoral joint contact force created by each muscle that spans the knee must be split between these lateral and medial compartments. This cannot be achieved by a consideration of the force parts of the equation of motion alone. This provides a further reason why it is important to explicitly include the joint reaction forces within the moment parts of the equations of motion:

2.5 $$\begin{array}{rl} & \left(\begin{array}{cccccccc} {\hat{p}}_{1}^{1}\cdots {\hat{p}}_{M}^{1} & {\hat{p}}_{\mathrm{pt}}^{1} & {\hat{q}}_{1}^{1}\cdots {\hat{q}}_{N}^{1} & {-I}_{3\times 3} & E_{3\times 3} & E_{3\times 3} & E_{3\times 3} & E_{3\times 3}\\ {\hat{p}}_{1}^{2}\cdots {\hat{p}}_{M}^{2} & {\hat{p}}_{\mathrm{pt}}^{2} & {\hat{q}}_{1}^{2}\cdots {\hat{q}}_{N}^{2} & I_{3\times 3} & {-I}_{3\times 3} & {-I}_{3\times 3} & E_{3\times 3} & E_{3\times 3}\\ {\hat{p}}_{1}^{3}\cdots {\hat{p}}_{M}^{3} & {\hat{p}}_{\mathrm{pt}}^{3} & {\hat{q}}_{1}^{3}\cdots {\hat{q}}_{N}^{3} & E_{3\times 3} & I_{3\times 3} & I_{3\times 3} & -I_{3\times 3} & I_{3\times 3}\\ {{\hat{r}}_{1}^{1}\times \hat{p}}_{1}^{1}\cdots {{\hat{r}}_{M}^{1}\times \hat{p}}_{M}^{1} & {{\hat{r}}_{\mathrm{pt}}^{1}\times \hat{p}}_{\mathrm{pt}}^{1} & {{\hat{s}}_{1}^{1}\times \hat{q}}_{1}^{1}\cdots {{\hat{s}}_{N}^{1}\times \hat{q}}_{N}^{1} & E_{3\times 3} & E_{3\times 3} & E_{3\times 3} & E_{3\times 3} & E_{3\times 3}\\ {{\hat{r}}_{1}^{2}\times \hat{p}}_{1}^{2}\cdots {{\hat{r}}_{M}^{2}\times \hat{p}}_{M}^{2} & {{\hat{r}}_{\mathrm{pt}}^{2}\times \hat{p}}_{\mathrm{pt}}^{2} & {{\hat{s}}_{1}^{2}\times \hat{q}}_{1}^{2}\cdots {{\hat{s}}_{N}^{2}\times \hat{q}}_{N}^{2} & {\tilde{d}}^{2} & {-\tilde{h}}_{1}^{2} & {-\tilde{h}}_{2}^{2} & E_{3\times 3} & E_{3\times 3}\\ {{\hat{r}}_{1}^{3}\times \hat{p}}_{1}^{3}\cdots {{\hat{r}}_{M}^{3}\times \hat{p}}_{M}^{3} & {{\hat{r}}_{\mathrm{pt}}^{3}\times \hat{p}}_{\mathrm{pt}}^{3} & {{\hat{s}}_{1}^{3}\times \hat{q}}_{1}^{3}\cdots {s_{N}^{3}\times \hat{q}}_{N}^{3} & E_{3\times 3} & {\tilde{d}}_{1}^{3} & {\tilde{d}}_{2}^{3} & E_{3\times 3} & {\tilde{f}}^{3}\\ {\hat{p}}_{1}^{\mathrm{pat}}\cdots {\hat{p}}_{M}^{\mathrm{pat}} & {\hat{p}}_{\mathrm{pt}}^{\mathrm{pat}} & E_{3\times N} & E_{3\times 3} & E_{3\times 3} & E_{3\times 3} & E_{3\times 3} & {-I}_{3\times 3}\\ \rho_{1}\cdots \rho_{M} & -1 & E_{1\times N} & E_{1\times 3} & E_{1\times 3} & E_{1\times 3} & E_{1\times 3} & E_{1\times 3} \end{array}\right)\left(\begin{array}{c} F_{1}\\ \vdots \\ F_{M}\\ F_{\mathrm{pt}}\\ L_{1}\\ \vdots \\ L_{N}\\ {\hat{R}}^{1}\\ {\hat{R}}_{1}^{2}\\ {\hat{R}}_{2}^{2}\\ {\hat{R}}^{3}\\ {\hat{R}}^{\mathrm{pat}} \end{array}\right)\\ & \;=\left(\begin{array}{c} m^{1}({\hat{a}}^{1}-\hat{g})-{\hat{S}}^{0}\\ m^{2}({\hat{a}}^{2}-\hat{g})\\ m^{3}({\hat{a}}^{3}-\hat{g})\\ m^{1}{\hat{c}}^{1}\times ({\hat{a}}^{1}-\hat{g})+Y_{3\times 3}^{1}{\ddot{\hat{\varphi }}}^{1}+{\dot{\hat{\varphi }}}^{1}\times Y_{3\times 3}^{1}{\dot{\hat{\varphi }}}^{1}-{\hat{d}}^{1}\times {\hat{S}}^{0}-M^{0}\\ m^{2}{\hat{c}}^{2}\times ({\hat{a}}^{2}-\hat{g})+Y_{3\times 3}^{2}{\ddot{\hat{\varphi }}}^{2}+{\dot{\hat{\varphi }}}^{2}\times Y_{3\times 3}^{2}{\dot{\hat{\varphi }}}^{2}\\ m^{3}{\hat{c}}^{3}\times ({\hat{a}}^{3}-\hat{g})+Y_{3\times 3}^{3}{\ddot{\hat{\varphi }}}^{3}+{\dot{\hat{\varphi }}}^{3}\times Y_{3\times 3}^{3}{\dot{\hat{\varphi }}}^{3}\\ E_{3\times 1}\\ 0 \end{array}\right). \end{array}$$

For each case, the equations of motion were solved for each time point using an optimization approach that is based upon a cost function adapted from the work of Crowninshield & Brand [24] and Raikova [25] and that we have used previously [11] (equation (2.6); table 4). The optimization was solved using the optimization toolbox of Matlab® (R2103a; The Mathworks, Inc, 2013):

2.6 $$\min_{F_{i},L_{j}}J=\sum_{i=1}^{M}{\left(\frac{F_{i}}{F_{\max_{i}}}\right)}^{3}+\sum_{j=1}^{N}{\left(\frac{L_{i}}{L_{\max_{i}}}\right)}^{3}.$$

The force upper bound of each muscle ($F_{\max_{i}}$) was calculated from the data of Klein Horsman *et al.* [15]. Specifically, the cross-sectional area of each muscle element was doubled to account for the larger muscle mass of our young, athletic population and then multiplied by an assumed maximum muscle stress (3.139×10^5^ N m^−2^). [26] The force upper bounds of the ligaments ($L_{\max_{j}}$) were similar to our previous work [11] (table 5). It should be noted that the ligaments are simply modelled as tensile force actuators. That is, the calculated ligament forces are not derived from measurements of ligament strain. A detailed justification and analysis of this approach (including a discussion of limitations) is provided in our previous work [11].

**Table 5.** Upper bounds of the ligaments included in this study.

ligament	joint	upper bound (N)
iliofemoral ligament (anterior)	hip	850
iliofemoral ligament (lateral)	hip	850
pubofemoral ligament	hip	450
ischiofemoral ligament	hip	450
anterior cruciate ligament	knee	2000
posterior cruciate ligament	knee	4000
medial collateral ligament	knee	3000
lateral collateral ligament	knee	2000
oblique popliteal ligament	knee	1000
posterior tibiotalar ligament	ankle	850
tibiocalcaneal ligament	ankle	850
tibionavicular ligament	ankle	850
posterior talofibular ligament	ankle	850
calcaneofibular ligament	ankle	850

### 2.8 Experimental data

The specific data used in this study has also been previously described [13,27,28] and is the same data that was used in our previous work describing optimization approaches to inverse dynamics analysis [9–11] (this being considered an advantage as it makes comparison between studies much easier). To summarize, the data describe the performance of vertical jumps performed by a group of athletic males (*n*=12; mean age 27.1±4.3 years; mean mass 83.7±9.9 kg) who provided informed consent prior to the data collection process (the data collection was approved by the institutional review board of St Mary's University College). The position of retro-reflective markers placed upon key anatomical landmarks [29,30] was captured at 200 Hz using Vicon (Vicon MX System, Vicon Motion Systems Ltd, Oxford, UK) and ground reaction forces were recorded synchronously using a Kistler force plate (Kistler Type 9286AA, Kistler Instrumente AG, Winterthur, Switzerland). Both the position and force data were filtered using a fifth order Woltring filter [31] before it was input into the model. The filtered raw data are provided with this article as the electronic supplementary material.

### 2.9 Statistical analysis

The performance of each case was evaluated by comparing the predicted muscle forces to electromyography (EMG) envelopes, representing the muscular activations during vertical jumping. These envelopes were taken from the work of Rodacki *et al.* [32] (who generated these envelopes from the raw electromyograms by using the Myo-Dat v. 5.0 EMG analysis software (MIE Medical Research Ltd., Leeds, UK) with a second-order low pass filter of 6 Hz). First, the mean muscle force at each time point (relative to the time of take-off) was calculated in order to produce a composite curve of muscle forces for each case. The similarity of this curve to the EMG data were quantified by using a Geers' metric (equation (2.7)) [33], which it has been suggested is appropriate for comparing measured and experimental curves in biomechanics [34–36]. The Geers' metric provides separate estimations of the magnitude error (*M*, which is insensitive to differences in phase between the two curves) and the phase error (*P*, which is insensitive to differences in magnitude between the two curves):

2.7 $$M=\sqrt{\frac{V_{cc}}{V_{mm}}}-1,\;P=\frac{1}{\pi }\cos^{-1}\frac{V_{mc}}{\sqrt{V_{mm}V_{cc}}},$$

where

2.8 $$\left.\begin{array}{rl} V_{mm} & =\frac{1}{t_{2}-t_{1}}\int_{t_{1}}^{t_{2}}{m(t)}^{2}\;dt,\\ V_{cc} & =\frac{1}{t_{2}-t_{1}}\int_{t_{1}}^{t_{2}}{c(t)}^{2}\;dt,\\ V_{mc} & =\frac{1}{t_{2}-t_{1}}\int_{t_{1}}^{t_{2}}m(t)c(t)\;dt, \end{array}\right\}$$

and *m*(*t*) and *c*(*t*) are the measured and calculated waveforms, respectively.

## 3. Results

On average, the optimization was able to find a solution for 95.1±6.2% of the analysed frames. The lowest overall joint contact forces were found in case 4 (table 6). There was a marked similarity in the joint contact forces found in cases 2 and 3, and the ankle and total tibiofemoral joint contact forces found in case 1 were also similar to cases 2 and 3.

**Table 6.** Mean peak joint contact forces (mean ± s.d.) predicted for each case in this study (TFJ, tibiofemoral joint contact force; PFJ, patellofemoral joint contact force).

		knee				
case	ankle	lateral TFJ	medial TFJ	total TFJ	PFJ	hip
1	6.7±1.1			8.5±1.6		8.7±2.0
2	6.7±1.1			8.5±1.6	9.9±3.0	7.2±1.9
3	6.7±1.1			8.5±1.6	10.0±3.0	7.3±1.8
4	6.7±1.1	2.7±1.3	4.5±0.6	6.2±1.0	8.2±2.5	6.2±1.3
Cleather *et al.*[11]	9.0±1.6			7.4±2.1		5.5±1.0

**Figure 3.**
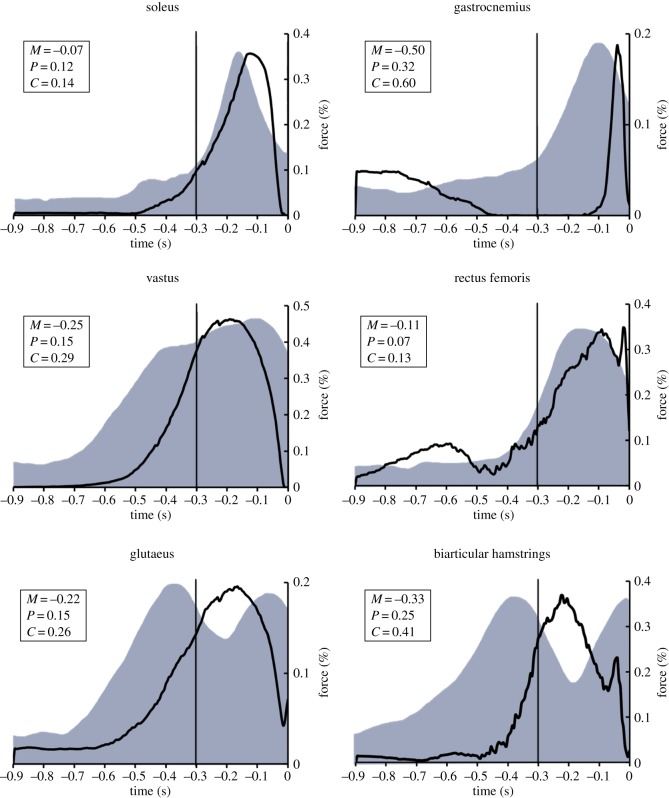
Average muscular recruitment during vertical jumping as predicted by case 4. The solid line represents the average percentage of maximum force capability expressed during jumping. The shaded grey regions represent EMG envelopes that have been reported previously in the literature (Rodacki *et al.* [32]). *M* is the magnitude error and *P* is the phase error (both taken from the Geers' metric).

There was a marked similarity between the predicted muscle forces and the EMG data across cases, as indicated by the similarity in the mean Geers' metrics (tables 7 and 8). For instance, figure 3 illustrates the mean muscle forces of six major muscle groups for case 4, in comparison to the EMG envelopes of Rodacki *et al.* [32]. There was a clear qualitative similarity between the model predictions and the EMG envelopes. The Geers' metric suggested a close agreement for soleus, rectus femoris, glutaeus and vastus, but demonstrated a lower level of agreement for gastrocnemius and the biarticular hamstrings.

**Table 7.** Magnitude error (Geers' metric) for the comparison of average predicted muscle forces with EMG envelopes for each case considered in this study (gastroc., gastrocnemius; r. fem., rectus femoris; bi. ham., biarticular hamstrings).

case	soleus	gastroc.	r. fem.	vastus	glutaeus	bi. ham.	mean ± s.d.
1	−0.05	−0.51	−0.11	−0.22	−0.19	−0.27	−0.22±0.14
2	−0.05	−0.49	−0.02	−0.25	−0.24	−0.30	−0.23±0.16
3	−0.08	−0.53	−0.08	−0.25	−0.24	−0.30	−0.25±0.15
4	−0.07	−0.50	−0.11	−0.25	−0.22	−0.33	−0.25±0.14

**Table 8.** Phase error (Geers' metric) for the comparison of average predicted muscle forces with EMG envelopes for each case considered in this study (gastroc., gastrocnemius; r. fem., rectus femoris; bi. ham., biarticular hamstrings).

case	soleus	gastroc.	r. fem.	vastus	glutaeus	bi. ham.	mean ± s.d.
1	0.11	0.33	0.07	0.14	0.13	0.21	0.16±0.09
2	0.11	0.34	0.04	0.15	0.15	0.22	0.17±0.09
3	0.12	0.34	0.05	0.16	0.15	0.23	0.17±0.09
4	0.12	0.32	0.07	0.15	0.15	0.25	0.18±0.08

## 4. Discussion

This study describes the development of a new musculoskeletal model of the lower limb that has been developed from our previous work in the estimation of muscle and joint contact forces during vertical jumping. The motivation for this was to create a musculoskeletal model that is segment-based. The results of this study demonstrate that the segment-based model has a similar ability to estimate muscle forces during vertical jumping as the previous approaches that we had described.

The performance of the model was assessed by comparing the muscle force estimations to muscular activations that had been previously reported within the literature. The mean muscle force estimations for each case were compared with the mean activation (EMG envelopes) using a Geers' metric, which provides values representing both the magnitude and phase errors. The differences between the estimated forces and the EMG envelopes showed only minor variations among cases, and the models demonstrated the closest match for soleus and rectus femoris, and the biggest discrepancies for gastrocnemius and the biarticular hamstrings (tables 7 and 8). Taken as a whole, the analysis presented here provides new and quantitative evidence as to the validity of this lower limb model.

The Geers' metric (phase error) demonstrated that the estimated muscle forces showed a consistent time lag relative to the EMG envelopes. Some degree of time lag would be expected because of the neuromechanical delay between muscular activation and force production, and so a zero phase error would not represent the best fit, and the phase error here is in the expected direction. Equally, the Geers' metric (magnitude error) demonstrated that the magnitudes of the model estimates were consistently lower than the muscular activations. A major factor in this trend was that the duration of peaks in estimated muscle forces was lower than the duration of the activations. It should be noted that muscle estimations and EMG envelopes that were compared in this study were not recorded concurrently, nor taken from the same subjects. Although this is a clear limitation of the study, and could account for some of the differences between the curves, there is evidence that the muscular activations during vertical jumping are remarkably stable between subjects and across jump conditions [32]. Therefore, given that the comparisons here are between group mean curves this was considered an acceptable limitation. In future work, we will seek to make the same comparisons at the subject level.

It is notable that the estimated biarticular muscle forces (biarticular hamstrings and gastrocnemius) showed a lower level of agreement than the monoarticular muscle force estimates. This was predominantly because of a later onset of muscular activation in the estimated muscle forces than was seen in the EMG envelopes. We have previously identified that modelling the function of the biarticular muscles is a key challenge for the musculoskeletal modelling community [9,10]. In particular, the benefits derived from the biarticular muscles transferring power between joints [37] may not be entirely captured by this modelling approach.

The modelling approach described in this paper is markedly different to other contemporary models of the lower limb. Many of these differences are consistent with, permitted by, or a consequence of the segment-based approach to biomechanics that is used here. Some of the more important differences and their ramifications are described below.

One trend within musculoskeletal modelling is to optimize the measured kinematics based upon kinematic constraints that describe the joints [7,12,38] (although there are other kinematic optimizations that do not assume joint constraints). One advantage of this is that these constraints can be used to limit the effects of measurement errors (for instance, soft tissue artefacts). However, the optimization in turn reduces the d.f. of the model. This may be disadvantageous, as these d.f. could be important in describing the mechanics of the limb. For instance, it is common to pose lower limb models which do not permit joint translations, however, we have recently demonstrated that the anterior–posterior translation of the tibiofemoral joint surfaces contributes to the ability of the musculature of the knee to rotate the thigh [5]. Equally, we have argued that it is more appropriate to retain the full amount of d.f. [39] and to instead represent the joints by modelling the forces that constrain the kinematic behaviour of the joint [1]. We believe that this is important as some of the structures that provide the forces that constrain the kinematics are those that are commonly injured.

The model that is described in this article does not impose such kinematic constraints. Instead, the position and orientation of each segment is determined independently. The potential problem of soft tissue artefacts is ameliorated by using a redundant number of markers on each segment, and then using the method of Horn [14] to find the best fit. All d.f. are consequently captured within this model, such that joint translations are permitted and all joints have three rotational d.f. The larger number of d.f. does bring its own costs however. In particular, the model presented here is not able to find a solution for all of the frames analysed. We believe this is a problem that would be eliminated as more detail is added to the model (by providing a greater number and variability in the lines of action of force to compensate for the greater number of d.f. [1,39–41]).

A common simplification in musculoskeletal modelling is the employment of multiple step solution processes that consider the equations of motion related to forces and moments independently [1,7,11]. The advantage of these approaches is that they reduce the complexity of the equations of motion, making their solution more straightforward. However, such an approach is not physiologically realistic—in the body forces and moments are equilibrated simultaneously and this dynamic interplay is important in understanding how the tissues of the body are loaded. In our previous work, we have described a one step, simultaneous inverse optimization approach which permits a more faithful representation of the function of biarticular muscles [9,10] and musculoligamentous interaction [11] during movement, and the same approach is employed in this model. Latterly, some other groups have adopted a similar approach and found it to be advantageous [7,12]. In particular, Moissenet *et al.* [7] have shown how such an approach can be used to successfully estimate the timing of musculotendon forces during gait. Equally, Hu *et al.* [12] found that such an approach predicted joint contact forces during gait that more closely matched the forces measured in patient populations than less complex (more traditional) approaches. Preliminary work therefore seems to suggest that more detailed modelling of the interplay of forces in creating linear and rotational motion is important to the understanding of movement.

One example of a contemporary multi-step approach is in the partitioning of tibiofemoral joint contact forces into medial and lateral components. For instance, Gerus *et al.* [23] describe a process whereby first the joint moments are calculated, second the muscle forces are determined based upon the calculated joint moments and then finally the joint contact forces are found such that the sum of the moments of muscle and joint contact forces is equal to the joint moment. This process is repeated twice, once for each of the lateral and medial compartments. However, this methodology has the same weaknesses as the multi-step approaches previously referred to. In particular, the medial and lateral tibiofemoral contact forces contribute to the equilibration of moments at the knee, and thus should be solved for simultaneously with the muscle and ligament forces. Only in this approach is the dynamic interplay that equilibrates forces and moments properly represented. In the model described here, the tibiofemoral joint contact forces are found simultaneously with the muscle and ligament forces (this represents a considerable advance over our previous work [9–11]).

When employing a joint-based, multi-step approach it is common to not include an explicit representation of the patellofemoral joint. This is an understandable limitation of the approach—if the lower limb is modelled as a chain of linked segments, and analysed in terms of the inter-segmental moments between those segments, it is difficult to include a consideration of the patella. However, in order to create a segment-based description of the lower limb it was necessary for us to develop a patella model and to include the patellofemoral joint contact force. The patella model incorporated within this model is simple—the movement of the patella is entirely determined by the flexion of the tibiofemoral joint, and this relationship is not changed to reflect subject-specific characteristics. The movement of the patella can have an important impact on the outputs of a musculoskeletal model, in large part due to its effect on the *P*/*Q* ratio and in turn the tension required in the quadriceps musculature. In our future work, we will develop the model described here to permit a subject-specific scaling of the patellofemoral model which, in particular, produces an accurate, subject-specific *P*/*Q* ratio.

To summarize, this paper has described the development of a segment-based model of the lower limb and has demonstrated that its muscle force estimates are in line with our previous work. The rationale for such a model was based on our recent theoretical work that demonstrated advantages of a segment-based approach [5,6]. However, this study has not sought to evaluate whether the segment-based model presented here has such advantages in comparison to more traditional joint-based models. One reason for this is that such a comparison is very difficult to make—there are a number of assumptions that are variable between the two approaches. Despite this, we intend to address this question in our future work, and the development of this model is an important step along the way.

Our future plans are not limited to the segment-/joint-based comparison. Work is already underway to try and validate the muscle force estimates of the model by comparison with directly measured experimental data and values from the literature [35]. We have also performed some preliminary work evaluating the ability of the model to detect clinically relevant differences in tibiofemoral joint contact forces following an exercise intervention [42]. In addition, we believe that the lack of understanding as to the behaviour of musculoskeletal models is a key impediment to the realization of a clinical tool [1,43]. It is our intention to perform a systematic, probabilistic analysis of the input parameters and assumptions behind FreeBody. This will be invaluable in guiding researchers towards those parameters which are most likely to require accurate subject-specific information, and the degree to which subject-specific accuracy is required. In addition, the statistical analysis will provide a robust estimate as to the potential error inherent to the current state of the art approaches.

## 5. Introducing FreeBody

Currently, the choice of musculoskeletal modelling software available for use is somewhat limited. The market is dominated by a small number of key products which include AnyBody (AnyBody Technology A/S, Aalborg, Denmark), SIMM (MusculoGraphics Inc., Santa Rosa, CA, USA), LifeModeler (LifeModeler Inc., San Clemente, CA, USA) and OpenSim (Simbios, Stanford, CA, USA). There is no doubt that these software packages represent the state of the art in musculoskeletal modelling software, especially given that they tend to be backed by some of the most illustrious research groups within the field (e.g. SIMM and OpenSim are associated with the research group of Scott Delp at Stanford University and AnyBody with John Rasmussen of Aalborg University). However, for the most part these packages are commercial concerns, and carry large licence fees which could be prohibitive for some users. One exception is the freely available OpenSim [44], which is used quite widely within the biomechanics community.

A further barrier to the wider adoption of musculoskeletal modelling software is that such packages tend to require a considerable time investment to learn about their operation and then to develop applications with the desired functionality. In particular, this will serve as a barrier to potential users who do not have a strong background in biomechanics, software programming, mathematics or physics. There is therefore a need for a musculoskeletal modelling application that is more user friendly and that makes this analysis approach available to a wider population of potential users.

The musculoskeletal model described in this article as case 4 is publicly available as both a Matlab application and in the original source code (at www.msksoftware.org.uk) and includes extensive documentation. In addition, the source code for the model and the Matlab application are provided with this article as the electronic supplementary material. The Matlab application is driven by a graphical user interface which makes the use of the model straightforward and intuitive and the ubiquity of Matlab within science and education means that many users will be very familiar with the model environment. We, therefore, hope that this version is almost entirely ‘plug and play’, that is that the user simply has to process their data into the appropriate input format, and then they can use the model as an analysis tool. It is our intention that this brings musculoskeletal modelling technology to an entirely new population of users. Our philosophy is also to create a research tool that has broad use for the widest possible range of users, and it is for this reason that we have released the underlying code. Ultimately, it is our hope that this release of FreeBody will help to encourage the development of technology within this area.

## Data accessibility

The raw data used in this study is provided with this article as the electronic supplementary material. The model that this article describes is available as a Matlab® application and in the original source code at www.msksoftware.org.uk. In addition, the source code for the model and the Matlab application are provided with this article as the electronic supplementary material.

## References

[1] Cleather DJ, Bull AM 2012 The development of lower limb musculoskeletal models with clinical relevance is dependent upon the fidelity of the mathematical description of the lower limb. Part 1: equations of motion. Proc. Inst. Mech. Eng. H 226, 120–13. (doi:10.1177/0954411911432104) 22468464 10.1177/0954411911432104

[2] Erdemir A, McLean S, Herzog W, van den Bogert AJ 2007 Model-based estimation of muscle forces exerted during movements. Clin. Biomech. 22, 131 154. (doi:10.1016/j.clinbiomech.2006.09.005) 10.1016/j.clinbiomech.2006.09.005 17070969

[3] Van den Bogert AJ, Schamhardt HC 1993 Multi-body modelling and simulation of animal locomotion. Acta Anat. (Basel) 146, 95 10. (doi:10.1159/000147428) 8470472 10.1159/000147428

[4] Blemker SS, Asakawa DS, Gold GE, Delp SL 2007 Image-based musculoskeletal modeling: applications, advances, and future opportunities. J. Magn. Reson. Imaging 25, 441 451. (doi:10.1002/jmri.20805) 17260405 10.1002/jmri.20805

[5] Cleather DJ, Southgate DFL, Bull AMJ 2014 On the role of the patella, ACL and joint contact forces in the extension of the knee. PLoS ONE 9, 115670 (doi:10.1371/journal.pone.0115670) 10.1371/journal.pone.0115670 PMC4275277 25536067

[6] Cleather DJ, Southgate DFL, Bull AMJ 2015 The role of the biarticular hamstrings and gastrocnemius muscles in closed chain lower limb extension. J. Theor. Biol. 365, 217 225. (doi:10.1016/j.jtbi.2014.10.020) 25451963 10.1016/j.jtbi.2014.10.020

[7] Moissenet F, Chèze L, Dumas R 2014 A 3D lower limb musculoskeletal model for simultaneous estimation of musculo-tendon, joint contact, ligament and bone forces during gait. J. Biomech. 47, 50 58. (doi:10.1016/j.jbiomech.2013.10.015) 24210475 10.1016/j.jbiomech.2013.10.015

[8] Pennestrì E, Stefanelli R, Valentini PP, Vita L 2007 Virtual musculo-skeletal model for the biomechanical analysis of the upper limb. J. Biomech. 40, 1350 1361. (doi:10.1016/j.jbiomech.2006.05.013) 16824531 10.1016/j.jbiomech.2006.05.013

[9] Cleather DJ, Goodwin JE, Bull AMJ 2011 An optimization approach to inverse dynamics provides insight as to the function of the biarticular muscles during vertical jumping. Ann. Biomed. Eng. 39, 147 160. (doi:10.1007/s10439-010-0161-9) 20862546 10.1007/s10439-010-0161-9

[10] Cleather DJ, Goodwin JE, Bull AMJ 2011 Erratum to: an optimization approach to inverse dynamics provides insight as to the function of the biarticular muscles during vertical jumping. Ann. Biomed. Eng. 39, 2476 2478. (doi:10.1007/s10439-011-0340-3) 10.1007/s10439-010-0161-9 20862546

[11] Cleather DJ, Bull AMJ 2011 An optimization-based simultaneous approach to the determination of muscular, ligamentous, and joint contact forces provides insight into musculoligamentous interaction. Ann. Biomed. Eng. 39, 1925 1934. (doi:10.1007/s10439-011-0303-8) 21445690 10.1007/s10439-011-0303-8

[12] Hu C-C, Lu T-W, Chen S-C 2013 Influence of model complexity and problem formulation on the forces in the knee calculated using optimization methods. Biomed. Eng. Online 12, 20 (doi:10.1186/1475-925X-12-20) 23496903 10.1186/1475-925X-12-20 PMC3606467

[13] Cleather DJ, Bull AMJ 2010 Lower-extremity musculoskeletal geometry affects the calculation of patellofemoral forces in vertical jumping and weightlifting. Proc. Inst. Mech. Eng. H 224, 1073–1083. (doi:10.1243/09544119JEIM731) 21053772 10.1243/09544119JEIM731

[14] Horn BKP 1987 Closed form solution of absolute orientation using unit quaternions. J. Opt. Soc. Am. A 4, 629 64. (doi:10.1364/JOSAA.4.000629)

[15] Klein Horsman MD, Koopman HFJM, van der Helm FCT, Poliacu Prose L, Veeger HEJ 2007 Morphological muscle and joint parameters for musculoskeletal modelling of the lower extremity. Clin. Biomech. 22, 239 247. (doi:10.1016/j.clinbiomech.2006.10.003) 10.1016/j.clinbiomech.2006.10.003 17134801

[16] Kobayashi K, Sakamoto M, Hosseini A, Rubash HE, Li G 2012 In-vivo patellar tendon kinematics during weight-bearing deep knee flexion. J. Orthop. Res. 30, 1596 1603. (doi:10.1002/jor.22126) 22492400 10.1002/jor.22126

[17] Nha KW, Papannagari R, Gill TJ, Van de Velde SK, Freiberg AA, Rubash HE, Li G 2008 In vivo patellar tracking: clinical motions and patellofemoral indices. J. Orthop. Res. 26, 1067 1074. (doi:10.1002/jor.20554) 18327809 10.1002/jor.20554 PMC3740383

[18] Charlton IW, Johnson GR 2001 Application of spherical and cylindrical wrapping algorithms in a musculoskeletal model of the upper limb. J. Biomech. 34, 1209 1216. (doi:10.1016/S0021-9290(01)00074-4) 11506792 10.1016/s0021-9290(01)00074-4

[19] Modenese L, Phillips ATM, Bull AMJ 2011 An open source lower limb model: hip joint validation. J. Biomech. 44, 2185 2193. (doi:10.1016/j.jbiomech.2011.06.019) 21742331 10.1016/j.jbiomech.2011.06.019

[20] De Leva P 1996 Adjustments to Zatsiorsky—Seluyanov's segment inertia parameters. J. Biomech. 29, 1223 1230. (doi:10.1016/0021-9290(95)00178-6) 8872282 10.1016/0021-9290(95)00178-6

[21] Dumas R, Aissaoui R, de Guise JA 2004 A 3D generic inverse dynamic method using wrench notation and quaternion algebra. Comput. Methods Biomech. 7, 159 166. (doi:10.1080/10255840410001727805) 10.1080/10255840410001727805 15512759

[22] Buff H-U, Jones LC, Hungerford DS 1988 Experimental determination of forces transmitted through the patello-femoral joint. J. Biomech. 21, 17 23. (doi:10.1016/0021-9290(88)90187-X) 3339023 10.1016/0021-9290(88)90187-x

[23] Gerus P, Sartori M, Besier TF, Fregly BJ, Delp SL, Banks SA, Pandy MG, D'Lima DD, Lloyd DG 2013 Subject-specific knee joint geometry improves predictions of medial tibiofemoral contact forces. J. Biomech. 46, 2778 2786. (doi:10.1016/j.jbiomech.2013.09.005) 24074941 10.1016/j.jbiomech.2013.09.005 PMC3888900

[24] Crowninshield RD, Brand RA 1981 A physiologically based criterion of muscle force prediction in locomotion. J. Biomech. 14, 793 801. (doi:10.1016/0021-9290(81)90035-X) 7334039 10.1016/0021-9290(81)90035-x

[25] Raikova RT 2009 Investigation of the influence of the elbow joint reaction on the predicted muscle forces using different optimization functions. J. Musculoskelet. Res. 12, 31 43. (doi:10.1142/S021895770900216X)

[26] Yamaguchi GT 2001 Dynamic modeling of musculoskeletal motion: a vectorized approach for biomechanical analysis in three dimensions. New York, NY: Springer.

[27] Cleather DJ, Goodwin JE, Bull AMJ 2013 Hip and knee joint loading during vertical jumping and push jerking. Clin. Biomech. 28, 98 103. (doi:10.1016/j.clinbiomech.2012.10.006) 10.1016/j.clinbiomech.2012.10.006 PMC3966561 23146164

[28] Cleather DJ, Goodwin JE, Bull AMJ 2013 Inter-segmental moment analysis characterises the partial correspondence of jumping and jerking. J. Strength Cond. Res. 27, 89 100. (doi:10.1519/JSC.0b013e31825037ee) 22362089 10.1519/JSC.0b013e31825037ee PMC3966562

[29] Van Sint Jan S 2005 Skeletal landmark definitions: guidelines for accurate and reproducible palpation. Report by the Department of Anatomy, University of Brussels, Brussels, Belgium. See http://www.ulb.ac.be/~anatemb

[30] Van Sint Jan S, Croce UD 2005 Identifying the location of human skeletal landmarks: why standardized definitions are necessary—a proposal. Clin. Biomech. 20, 659 660. (doi:10.1016/j.clinbiomech.2005.02.002) 10.1016/j.clinbiomech.2005.02.002 15927740

[31] Woltring HJ 1986 A Fortran package for generalized, cross-validatory spline smoothing and differentiation. Adv. Eng. Softw. 8, 104 113. (doi:10.1016/0141-1195(86)90098-7)

[32] Rodacki AL, Fowler NE, Bennett SJ 2002 Vertical jump coordination: fatigue effects. Med. Sci. Sports Exerc. 34, 105 116. (doi:10.1097/00005768-200201000-00017) 11782655 10.1097/00005768-200201000-00017

[33] Geers TL 1984 An objective error measure for the comparison of calculated and measured transient response histories. Shock Vib. Bull. 54, 99 107

[34] Lund ME, de Zee M, Rasmussen J 2011 Comparing calculated and measured curves in validation of musculoskeletal models. In XIII Int. Symp. on Computer Simulation in Biomechanics, Leuven, Belgium.

[35] Price PDB, Gissane C, Cleather DJ 2014 The validation of a 3D lower limb musculoskeletal model. In Bioengineering14, Imperial College London, UK.

[36] Lund ME, de Zee M, Andersen MS, Rasmussen J 2012 On validation of multibody musculoskeletal models. Proc. Inst. Mech. Eng. H 226, 82–94. (doi:10.1177/0954411911431516) 22468460 10.1177/0954411911431516

[37] Bobbert MF, van Ingen Schenau GJ 1988 Coordination in vertical jumping. J. Biomech. 21, 249 26. (doi:10.1016/0021-9290(88)90175-3) 3379084 10.1016/0021-9290(88)90175-3

[38] Prinold JA, Masjedi M, Johnson GR, Bull AM 2013 Musculoskeletal shoulder models: a technical review and proposals for research foci. Proc. Inst. Mech. Eng. H 227, 1041–1057. (doi:10.1177/0954411913492303) 23851656 10.1177/0954411913492303

[39] Cleather DJ, Bull AMJ 2011 Knee and hip joint forces: sensitivity to the degrees of freedom classification at the knee. Proc. Inst. Mech. Eng. H 225, 621–626. (doi:10.1177/0954411911399975) 22034745 10.1177/0954411911399975

[40] Glitsch U, Baumann W 1997 The three-dimensional determination of internal loads in the lower extremity. J. Biomech. 30, 1123 1131. (doi:10.1016/S0021-9290(97)00089-4) 9456380 10.1016/s0021-9290(97)00089-4

[41] Dumas R, Moissenet F, Gasparutto X, Cheze L 2012 Influence of joint models on lower-limb musculo-tendon forces and three-dimensional joint reaction forces during gait. Proc. Inst. Mech. Eng. H 226, 146–160. (doi:10.1177/0954411911431396) 22468466 10.1177/0954411911431396

[42] Czasche MB, Goodwin JE, Bull AMJ, Cleather DJ Submitted The effects of an eight week strength training intervention on tibiofemoral joint loading during landing. 10.1136/bmjsem-2017-000273 PMC5783107 29387442

[43] Cleather DJ, Bull AM 2012 The development of lower limb musculoskeletal models with clinical relevance is dependent upon the fidelity of the mathematical description of the lower limb. Part 2: patient-specific geometry. Proc. Inst. Mech. Eng. H 226, 133–145. (doi:10.1177/0954411911432105) 22468465 10.1177/0954411911432105

[44] Delp SL, Anderson FC, Arnold AS, Loan P, Habib A, John CT, Guendelman E, Thelen DG 2007 OpenSim: open-source software to create and analyze dynamic simulations of movement. IEEE Trans. Biomed. Eng. 54, 1940 1950. (doi:10.1109/TBME.2007.901024) 18018689 10.1109/TBME.2007.901024

